# Microbial community analysis of apple rhizosphere around Bohai Gulf

**DOI:** 10.1038/s41598-017-08398-9

**Published:** 2017-08-21

**Authors:** Jihang Jiang, Zhen Song, Xiaotong Yang, Zhiquan Mao, Xiaohong Nie, Hui Guo, Xiawei Peng

**Affiliations:** 1 College of Biological Sciences and Biotechnology, Beijing Forestry University, Beijing, 100083 China; 2 Institute of Environment and Sustainable Development in Agriculture, Chinese Academy of Agricultural Sciences, Beijing, 100081 China; 3 College of Horticulture Science and Engineering, Shandong Agricultural University, Taian, 271000 China

## Abstract

Bohai Gulf is the main area for apple tree cultivation in China. Consecutive replanting significantly affects the yield and quality of apple trees in this area. Microecological imbalance in apple trees’ rhizospheres caused by variation in the soil microbial community is considered the primary cause of apple replant disease (ARD). This study analysed the microbial communities of the rhizospheres of perennial apple trees (PAT) and apple tree saplings under replanting (ATS) around Bohai Gulf using high-throughput sequencing. The results revealed increased populations of typical pathogenic fungi *Verticillium* and bacteria *Xanthomonadaceae*, and decreased populations of beneficial bacterial populations *Pseudomonas* and *Bacillus* with replanting, suggesting that competition between pathogens and beneficial microbes varies according to the ratio of pathogens to beneficial microbes in rhizosphere soil under the replanting system. Meanwhile, replanting was accompanied by an increase in the antagonistic bacteria *Arthrobacter* and fungus *Chaetomium*, suggesting that increased numbers of pathogens can lead to more instances of antagonism. Redundancy analysis (RDA) revealed site position and the main soil properties (pH, organic matter, available N, available K, available P, and moisture) affected the microbial community composition. It found clear differences in soil microbial communities and demonstrated a better understanding of the causes for ARD.

## Introduction

Apple replant disease (ARD) is common in all major apple growing areas in the world. ARD has been linked to a substantial decrease in both yield and quality of apples in most apple growing regions, with economic losses equivalent to as much as 50% of production throughout the lifetime of the apple orchard^1^. The etiology of this disease has been reported over the past few decades in North America and Europe, as well as in many other parts of the world, including South Africa, China, New Zealand, and Tasmania^2^.

However, the etiology of ARD remains unclear. Various factors, both biotic and abiotic, have been linked to the induction of ARD^3^. Recently, many studies have speculated that disruption of the soil microbial community by long-term replanting significantly contributes to ARD^4^. Previous studies using soil fumigation, soil pasteurisation^2, 5, 6^, and soil sterilisation have shown significantly increased apple tree growth in treated soils compared with untreated soils, which indicates that microbial communities play an important role in apple tree health^2^. The aboveground performance of plants is closely correlated with changes in underground microbial communities^7^. Soil ecosystem function is governed largely by the microbial dynamics of the rhizosphere, as microbial community composition and diversity affect soil structure and biological interactions^8^.

The majority of apple trees are planted in Bohai Gulf, the epicentre of China’s apple tree cultivation. Here consecutive replanting significantly affects the yield and quality of apple trees, causing heavy economic losses. As replanting is commonly linked to the increased inoculum and activity of soil-borne plant pathogens, as well as the disruption of the soil microbial community^6, 9–11^, it has long been recognised as a cause of reduced yields over time in other species. However, it has not been linked to variation of the rhizosphere’s microbial communities of apple trees in Bohai Gulf. Meanwhile, numerous studies have shown that environmental factors shape community structure^12–15^. The composition of the microbial community’s structure greatly affects the interaction between environmental factors and microorganisms^7, 16^. In the present study, both bacterial and fungal communities in the apple tree’s rhizosphere of Bohai Gulf were investigated through high-throughput sequencing. Redundancy analysis was carried out so as to detect the abundance of microbial groups and the interaction between microbial groups and soil environmental properties. Our research compared the microbial community’s composition and structure by examining the presence of bacteria and fungi in perennial apple tree (PAT) and apple tree saplings under replanting (ATS) soils of Bohai Gulf to monitor variations after replanting. This was done to provide a basis for reasonable understanding of the rhizosphere microbial community’s role in overcoming ARD.

## Results

### Estimators of the diversity and species richness of microbial communities

All of the raw sequence data obtained was assigned to each sample based on their barcode sequence. A total of 623,094 raw bacterial reads and 524,083 raw fungal reads were obtained from the rhizosphere of PAT and ATS soils. After trimming the barcodes, adapters, and primers and filtering the low-quality sequences, 480,262 bacterial sequences (ranging from 34,291 to 59,734 sequences per sample) and 468,134 fungal sequences (ranging from 38,785 to 59,051 sequences per sample) were used for further analysis.

Bacterial 16S rRNA reads numbering 480,262 were obtained from ten sequencing samples with an average length of 438 bp; the number of each sample was as follows: 49,826 (A1); 51,166 (A2); 59,734 (B1); 41,386 (B2); 49,390 (C1); 47,240 (C2); 45,400 (D1); 54,440 (D2); 34,291 (E1); and 473,899 (E2). The majority of the read lengths was focused on 400~460 bp, accounting for 99.67%.

Fungal 18S rRNA yielded 468,134 reads in ten sequencing samples in total, low-quality and short sequence reads excluded, with an average length of 401 bp. The number of each sample was 40,773 (A1); 45,151 (A2); 52,708 (B1); 59,051 (B2); 40,409 (C1); 52,409 (C2); 38,785 (D1); 41,717 (D2); 51,379 (E1); and 45,752 (E2). The sequence length in 401~420 bp accounted for the largest proportion, which was 92.52%, and the reads within 341~400 bp accounted for 7.47% (ranked second). Based on 97% similarity, 145,188 and 136,015 OTUs were obtained for 16S rRNA and 205,039 and 222,131 OTUs were found for 18S rRNA, respectively, from PAT and ATS soils of five sites (Supplementary Fig. S1).

**Figure 1:**
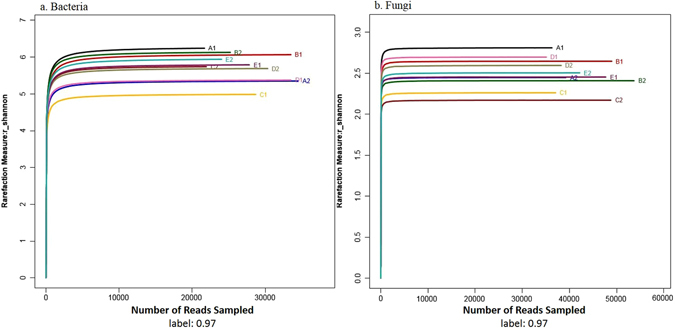
Shannon-Wiener curves of bacterial (**a**) and fungal (**b**) depicting the effect of 3% dissimilarity on the number of OTUs identified in the 10 soil samples. The “A1”, “B1”, “C1”, “D1” and “E1” refers to the five PAT soils in Qixia, Muping, Laizhou, Huludao and Changli. The “A2”, “B2”, “C2”, “D2” and “E2” refers to the five ATS soils in Qixia, Muping, Laizhou, Huludao and Changli, respectively.

Using a 3% dissimilarity cut-off for clustering, the reads were grouped into different OTUs. All chimera were removed during the process of clustering and the representative OTUs were obtained to classify the species. Rarefaction curves (Supplementary Fig. S2) for the microbial community at distance levels of 0.03 had not reached an asymptote, which indicated that the sequence was not sufficient to represent the different bacterial and fungal communities; however, by combining the rarefaction curves with the Shannon diversity index (Fig. 1), the Shannon diversity curves approached a plateau. Therefore, the data were sufficient to allow an analysis of microbial communities.

Soil available K (AK) and available P (AP) decreased significantly in ATS soils compared with PAT soils overall (Table 1). Available N (AN) increased in samples A, B, and C from the Shandong area and decreased in samples D and E. Compared with PAT soils, pH did not differ significantly in ATS soils. No statistically significant difference was observed in organic matter (OM) and moisture between PAT soils and ATS soils. The indices of diversity and richness of bacteria and fungi are shown in Table 2. The depth of all sample sequences was 98% indicating adequate sequence coverage, thus meeting the criteria of our experiments. The ACE and Chao values are indicators of species richness. The Shannon value is an indicator of microbial diversity. The Qixia sample showed the highest microbial community diversity in PAT soil for both bacteria and fungi. The results above showed that soil biodiversity and soil properties differed between sampling locations, and replanting did not have a significant overall effect on microbial diversity and richness.

**Table 1:** Physicochemical parameters of the soil samples.

Samples	Available N (%)	Organic matter g/kg	Available K mg/kg	Available P mg/kg	pH	Moisture %
PAT soils						
A1 Qixia	1150 ± 0.0055	15.4 ± 0.01	31457.25 ± 0.0038	966.27 ± 0.0087	5.91 ± 0.30	89.29 ± 0.002
B1 Muping	9560 ± 0.0091	10.1 ± 0.008	110716.12 ± 0.0028	248.27 ± 0.0046	5.345 ± 0.43	91.47 ± 0.0069
C1 Laizhou	1460 ± 0.0056	15.8 ± 0.0099	112304.99 ± 0.0082	722.69 ± 0.0042	5.28 ± 0.014	89.88 ± 0.0047
D1 Huludao	3210 ± 0.0046	12.1 ± 0.0095	30441.40 ± 0.0099	1162.99 ± 0.0063	5.265 ± 0.35	94.1 ± 0.0069
E1 Changli	1310 ± 0.0082	23.1 ± 0.0092	39949.37 ± 0.0082	1177.90 ± 0.0068	5.11 ± 0.11	88.02 ± 0.0054
ATS soils						
A2 Qixia	1230 ± 0.0036	11.8 ± 0.0027	14645.06 ± 0.0050	364.03 ± 0.0019	4.71 ± 0.071	90.82 ± 0.0071
B2 Muping	1140 ± 0.0046	20.5 ± 0.015	107702.27 ± 0.0079	374.87 ± 0.0033	8.69 ± 1.54	86.845 ± 0.0023
C2 Laizhou	1610 ± 0.0053	14 ± 0.0246	88604.83 ± 0.0052	387.11 ± 0.0058	6.755 ± 0.42	87.71 ± 0.0013
D2 Huludao	1320 ± 0.0087	13.4 ± 0.008	28661.68 ± .0061	399.01 ± 0.0064	5.56 ± 0.028	91.52 ± 0.0028
E2 Changli	930 ± 0.0108	28.6 ± 0.0099	14157.76 ± 0.0064	1170.640273 ± 0.0043	6.6 ± 0.40	87.205 ± 0.0011

**Table 2:** Number of sequences analysed, observed diversity richness (OTUs) and diversity/richness indices of the 16S rRNA bacterial and 18S rRNA fungal libraries obtained for clustering at 97% identity.

Sites	Location	Sample ID	ACE	Chao	Shannon	Coverage
Bacteria						
Qixia	37.28N	A1	1580.31	1617.61	6.24	98.76
	120.83E	A2	1360.99	1388.38	5.35	99.26
Muping	37.38N	B1	1653.55	1669.03	6.07	99.32
	121.59E	B2	1384.89	1426.26	6.12	99.14
Laizhou	37.18N	C1	989.30	1001.07	4.98	99.38
	119.93E	C2	1132.46	1156.59	5.73	99.16
Huludao	40.72N	D1	1105.24	1111.96	5.37	99.41
	120.83E	D2	1195.41	1216.47	5.68	99.37
Changli	39.70N	E1	1253.25	1301.77	5.79	99.32
	119.17E	E2	1385.49	1418.75	5.93	99.04
Fungi						
Qixia	37.28N	A1	113.16	119.50	2.81	99.96
	120.83E	A2	89.611	85.50	2.45	99.97
Muping	37.38N	B1	95.20	93.67	2.64	99.99
	121.59E	B2	117.34	123.43	2.41	99.96
Laizhou	37.18N	C1	84.58	83.88	2.26	99.97
	119.93E	C2	85.83	86.43	2.17	99.98
Huludao	40.72N	D1	82.59	82.67	2.70	99.98
	120.83E	D2	83.15	82.43	2.60	99.99
Changli	39.70N	E1	94.48	94.00	2.45	99.99
	119.17E	E2	101.53	100.63	2.50	99.98

### Soil community composition and structure analysis

There were 2,126 bacterial OTUs obtained from the ten samples, as classified by the MOTHUR program; these were then clustered into 31 bacterial phyla. When lumping the OTUs at the phylum level of bacteria, no significant differences were observed between PAT and ATS soils (Fig. S3). Proteobacteria, Actinobacteria, Acidobacteria, Chloroflexi, Firmicutes, and Gemmatimonadetes were dominant phyla existing in all samples, with Proteobacteria representing the most dominant phylum and accounting for 39.83% (A1), 30.27% (B1), 45.63% (C1), and 38.59% (E1) in PAT soils and 37.32% (A2), 31.79% (B2), 55.83% (C2), and 49.27% (E2) in ATS soils. Acidobacteria were the second-most prevalent, accounting for 15.37% (A1), 15.90% (B1), 14.89% (C1), and 13.18% (E1) in PAT soils and 24.20% (A2), 13.38% (B2), 9.71% (C2), and 14.22% (E2) in ATS soils. In contrast, Acidobacteria was the dominant phylum in both PAT (29.18%) and ATS (25.96%) soils while Proteobacteria was less prevalent in sample D, accounting for 15.59% (D1) and 25.26% (D2). Bacteroidetes, Nitrospirae, Cyanobacteria, and Verrucomicrobia were also present in all samples but with low richness.

Similarly with bacteria, there was no significant difference among fungi at the phylum level between PAT and ATS soils. There were 169 fungal OTUs obtained from the ten samples; the MOTHUR program was used to classify the OTUs, which were clustered into 29 fungal phyla. Ascomycota, Basidiomycota, Mucoromycotina, Ciliophora, and Glomeromycota were the dominant phyla across all samples, with Ascomycota representing the most dominant phylum and accounting for 82.44% (A1), 84.63% (B1), 73.80% (C1), 79.63% (D1), and 89.54% (E1) in PAT soils and 91.25% (A2), 88.82% (B2), 94.71% (C2), 68.69% (D2), and 76.29% (E2) in ATS soils.

### Comparison of microbial communities from PAT and ATS soils

**Figure 2:**
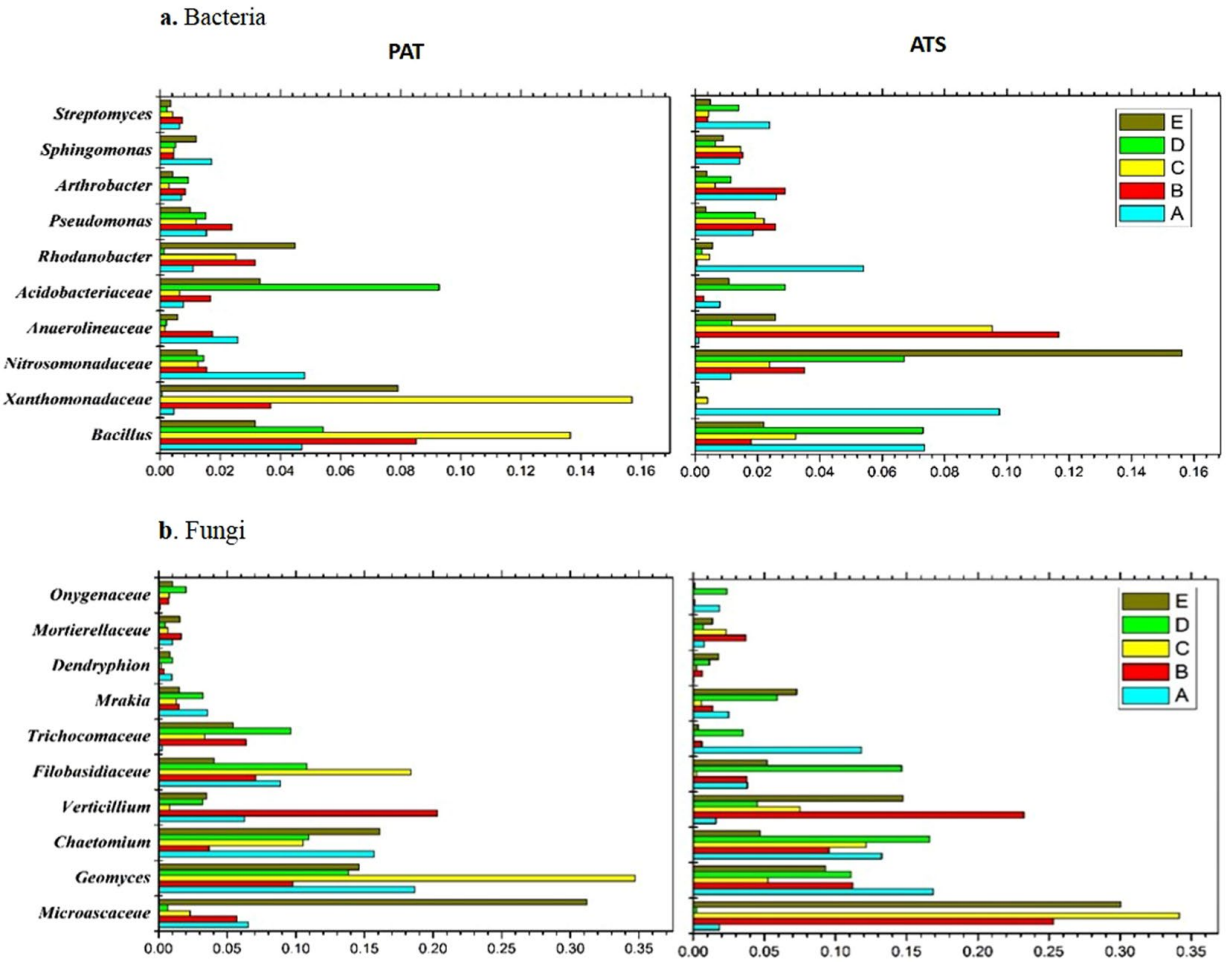
Relative abundances of the dominant bacterial (**a**) and fungal (**b**) groups in PAT and ATS rhizosphere soil. The “A”, “B”, “C”, “D” and “E” refers to the five sampling sites: Qixia, Muping, Laizhou, Huludao and Changli, respectively.

The relative abundance of different bacterial and fungal groups from the rhizosphere was compared (Fig. 2). For bacteria, the most abundant OTUs belonged to *Bacillus*, *Xanthomonadaceae*, and *Nitrosomonadacea*. By exploring the composition of the ten samples, *Bacillus* was found to be the dominant group in both PAT and ATS soils. Compared with PAT soils, *Bacillus* was present in relatively smaller quantities in ATS soils. *Xanthomonadaceae* had relatively greater population size in ATS soils, especially in Qixia. *Nitrosomonadaceae* was found in relatively higher amounts in ATS soils from all five sites, and the abundance of *Arthrobacter* and *Pseudomonas* in ATS samples was also relatively high from four sites with the exception of Changli. For fungi, the most abundant 10 OTUs accounted for 67.63% of the total number of sequences, belonging to *Microascaceae*, *Geomyces*, *Chaetomium*, *Verticillium*, and *Filobasidiaceae. Microascaceae*, *Filobasidiaceae*, and *Verticillium* were higher in ATS soils than in PAT soils overall, while *Geomyces* and *Chaetomium* were relatively less, especially in Qixia and Changli.

### Relationship between microbial community and environmental variables

**Figure 3:**
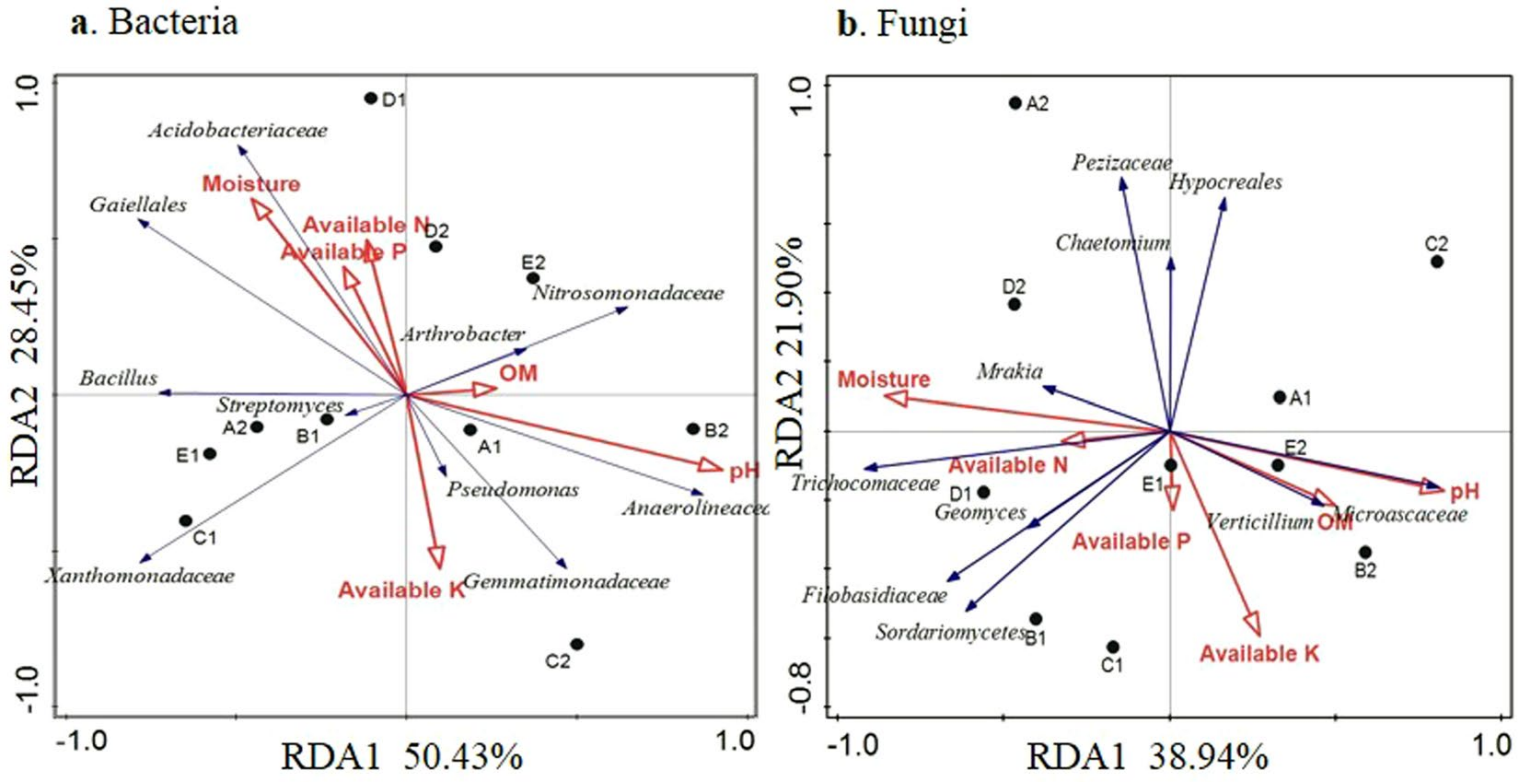
Redundancy analysis of abundant bacterial (**a**) and fungal (**b**) genus and soil properties for PAT and ATS samples from 5 sampling areas. The “A1”, “B1”, “C1”, “D1” and “E1” refers to the five PAT soils in Qixia, Muping, Laizhou, Huludao and Changli. The “A2”, “B2”, “C2”, “D2” and “E2” refers to the five ATS soils in Qixia, Muping, Laizhou, Huludao and Changli, respectively.

Redundancy analysis showed that the relative abundance of bacteria was affected both by growing positions and soil properties (Fig. 3). The abundance of bacteria in D1 and D2 correlated with moisture, AN, and AP, while the abundance of bacteria in both E1 and E2 had no significant correlation with soil properties. These results suggest that the abundance of bacteria of two treatments in D and E had no obvious difference. However, the bacteria abundance in A1 correlated positively with pH and OM, while the abundance in A2 correlated negatively with pH and OM. Oppositely, the abundance in B1 correlated negatively with pH and OM while the abundance in B2 had no significant correlation with pH and OM. The abundance in C2 correlated with AK; the abundance in C1 presented no significant correlation with soil properties. Thus, replanting displayed a significant effect on microbial abundance, especially in areas of Shandong, and less of an effect on other areas around Bohai Gulf.

Redundancy analysis showed that the first and second components explained 78.88% of the total bacterial fungal variations. For bacteria, the first component of the redundancy analysis (RDA1), which explained 50.43% of the total variation, separated PAT soil samples obtained from the five sites from ATS soil samples. Soil OM had a positive effect on *Nitrosomonadaceae* and *Arthrobacter*. Soil pH had a positive effect on *Anaerolineaceae*. Soil moisture and the content of AN and AP had a positive effect on *Acidobacteriaceae*. AK had a positive effect on *Pesudomonas* and *Gemmatimonadaceae*. Thus, soil properties, especially pH and OM influence the abundance of beneficial soil microbes.

Similar to bacterial abundance, the relative fungal abundance was affected by growing positions and soil properties (Fig. 3). Redundancy analysis showed that the first and second RDA components explained 60.84% of the total fungal variations. The abundance of fungi in A1 correlated positively with pH, while the abundance of A2 had an opposite relationship with pH. The abundance in B1 correlated positively with the content of AP and AN while the abundance in B2 correlated positively with pH and OM. AP correlated positively with the abundance of C1 while correlating negatively with the abundance of C2. Environmental variables had less of an effect on the abundance of D and E, indicating that replanting had a greater impact on the fungal community in QiXia, LaiZhou, and Muping, all of which are located in the Shandong area and not in the northern areas in Bohai Gulf. *Trichocomaceae* significantly correlated with AN, as pH had a positive effect on *Microascaceae*. OM had a positive effect on *Verticillium*, indicating that the content of OM can reflect the abundance of pathogen *Verticillium*. *Filobasidiaceae* correlated positively with AN.

## Discussion

Soil biological activity can be measured through the changes in microbial quantity and species. It is considered that imbalance of the soil microbial community is an important contributor to replant disease. Microbes can affect the growth of crops by affecting the physical and chemical properties of soil and the transportation of nutrients needed for the cultivation of crops. In this study, Illumina MiSeq sequencing analysis of 16S rRNA and 18S rRNA gene sequences revealed that replanting altered rhizospheric microbe diversity and the community structure of apple trees in the Central Bohai area.

In this study, no significant differences among the OTUs were observed at the phylum level between ATS and PAT soils, both in bacteria and fungi. This result is in accordance with the findings by Xu *et al*. and Jumpponen and Jones, who also investigated soil fungal communities using deep amplicon sequencing. According to the diversity and richness index of the rhizosphere soil, no overall effect was observed in PAT soils compared to ATS soils.

Further study at the level of the family and genus was conducted. Comparisons of PAT and ATS soils revealed differences in the rhizosphere resident bacterial genus. Latz *et al*. suggested that plant diversity improved protection against soil-borne pathogens by fostering antagonistic bacterial communities, while plant monocultures had a negative impact on the abundance of antagonistic microbes. The present study found an abundance of fungal OTUs belonging to *Verticillium*, which causes wilt or root rot in many plants^17^. The proportion of *Verticillium* in ATS soils of B, C, D and E increased 2.89%, 6.73%, 1.31%, and 11.29%, respectively, compared with PAT soils. Its relative abundance significantly increased with continuous farming of apples, suggesting that this pathogen may be the causal agent of ARD in our sample fields. Similarly, when crops such as winter rapeseed and cotton were replanted, a significant increase in the abundance of *Verticillium* in soil and aggravated root wilt was observed^18^. Previous results have indicated that replanting contributes to the growth of harmful fungi *Verticillium* ^9, 11, 19^, similar to the results shown in this study. *Xanthomonadaceae* has been reported as a cotton pathogen linked to decline in cotton growth and soil suppressiveness in replanted soils, also observed in ATS soils^9, 20^. *Bacillus* showed a decline in ATS soils in this study. As reported, it is beneficial for the growth of black pepper, zea, Spartina maritima, and wheat^7^; additionally, *Bacillus* spp. is known to be a plant-beneficial rhizobacteria with the potential to combat phytopathogens through the release of active secondary metabolites. In accordance with our previous study results, compared with PAT soils, the microbial community in the rhizosphere of ATS soils was more conducive to pathogenic microorganisms and less beneficial to microorganisms, which may be the root cause of poor growth of apple trees in ATS soils.

It is worth noting that some beneficial microbes, such as *Arthrobacter* and *Chaetomium*, presented an increasing trend in ATS soils in Muping, Laizhou, and Huludao. *Arthrobacter* act as antagonistic bacteria against *Sclerotinia sclerotiorum* ^21^, which can cause sunflower sclerotinia rot^22^. *Arthrobacter* also show a significant negative correlation with tobacco bacterial wilt disease^23^. The proportion of *Arthrobacter* bacteria has had significantly increased in ATS soils of the Bohai Gulf. *Chaetomium* can be used for controlling disease in other plants^24^. By acting as a biocontrol fungi against plant pathogenic bacteria, it also has the ability to decompose palm-oil mill fibres^25^. *Nitrosomonadaceae* and *Pseudomonas* act as beneficial bacteria and also present an increasing trend in ATS soils. A previous study found that the addition of *Nitrosomonadaceae* could reduce nitrogen loss, as well as the time required to stabilise the nitrogen profile^26^. Additionally, *Nitrosomonadaceae* is useful for biotechnological processes such as bioremediation of toxic chemicals in the soil^27^. High-throughput sequencing in this study confirmed an increase in the abundance of *Pseudomonas*. Root-associated *Pseudomonas* species are well known to produce metabolites that can limit the growth of other microorganisms^28^. There are reports of both beneficial and harmful species of *Pseudomonas* ^29–32^, however, the majority of previous studies have found that *Pseudomonas* spp. have the potential to control specialised pathogens and suppress soil-borne diseases^30, 31^. The increase of these beneficial microbes in ATS soils may suggest that populations are more competitive than other species, allowing them to prevail in ATS soils. A reasonable explanation of the above results is that beneficial microbes existing in soil may act as antagonists inhibiting the harmful microbes. Therefore, the larger population of pathogens, e.g. *Verticillium* in ATS soils, may result in more intense competition among some antagonistic microbes. Thus, promoting beneficial microbes acting as antagonists may warrant further investigation.

Above all, competition, antagonism, and hyperparasitism are generally the main interactive mechanisms between pathogenic fungi and antagonists. Some beneficial microorganisms act as potential antagonists of plant pathogens present in the soil. Many previous findings have demonstrated that when attacked by a root pathogen, plants can alter their specific rhizosphere microbial populations for protection^33^. Thus, our results suggest that the reduction of apple growth in ATS soils may arise from the decline in soil populations of beneficial microorganisms and an increase of pathogens in ATS soils.

Soil microorganisms not only have mutual effects on each other but also interact with the surrounding environment. Mounting evidence indicates that root exudates initiate and modulate the dialogue between the plant and soil microbes, composed of both pathogenic and beneficial microbes^34^. With the replanting of apple trees, the characteristics of rhizosphere soil changes, causing shifts in the characteristics of root exudates due to apple physiological changes. This may systemically affect interactions between plants and microbes by decreasing the abundance of rhizosphere microorganisms’ taxa. In accordance with our previous study results, the soil AP and AN decreased significantly, that may have been caused by inhibition of the phosphate-solubilizing and potassium-solubilizing microbes’ activities. In this study, further understanding of the mechanism of microbial activity was described by redundancy analysis, revealing a significant correlation between soil properties and the abundance of some microbes. The apple tree’s growth position was associated with the abundance of soil microbes. Because the abundance of *Acidobacteriaceae* correlated significantly with soil moisture and AP, it was inferred that *Acidobacteriaceae* probably can dissolve phosphorus in the soil. The abundance of *Anaerolineaceae* significantly correlated with pH and soil OM. It was inferred that *Anaerolineaceae* may require particular pH levels; moreover, previous studies have found that anaerobic bacteria could promote the decomposition of OM in soil. For fungi, redundancy analysis indicated that the abundance of *Verticillium* had a positive correlation with OM. In future studies, it may be possible to control the content of the pathogenic *Verticillium* by manipulating the content of OM. The present study determined that different biocontrol microbes have different resistance mechanisms, and that some are affected by a single mechanism while others are affected by a variety of mechanisms. These findings suggest that with continued heavy apple cultivation, soil microbial communities may be transformed from a biological system suitable for apple tree growth to a biological system that promotes ARD.

## Conclusion

In conclusion, our research revealed that the ARD closely associates with the variation in micro-ecology in the rhizosphere’s ecosystem. High-throughput sequencing of PAT and ATS soils revealed that the bacterial and fungal community composition did not differ significantly at the phylum level. However, further analysis showed that the apple replanting system caused changes in the structure of the microbial community, ultimately resulting in the deterioration of microbial structures in rhizosphere soil, with fewer beneficial microorganisms and more pathogenic microbes. This imbalance in the rhizosphere’s microecological system leads to serious replanting problems in a continuous cropping system. Meanwhile, the antagonistic microbes play an important role in shaping the rhizosphere’s microbial community, as a greater population of pathogens in ATS soils may result in more intense competition by some antagonistic microbes which open several new options related to these newly discovered involvements in ARD outside of the relationship between microbial communities and environmental variables discussed in our research. This finding provides a clue to open a new avenue for modulating the root microbiome to enhance crop production and sustainability.

## Materials and Methods

### Sample selection and soil physiochemical properties

**Figure 4:**
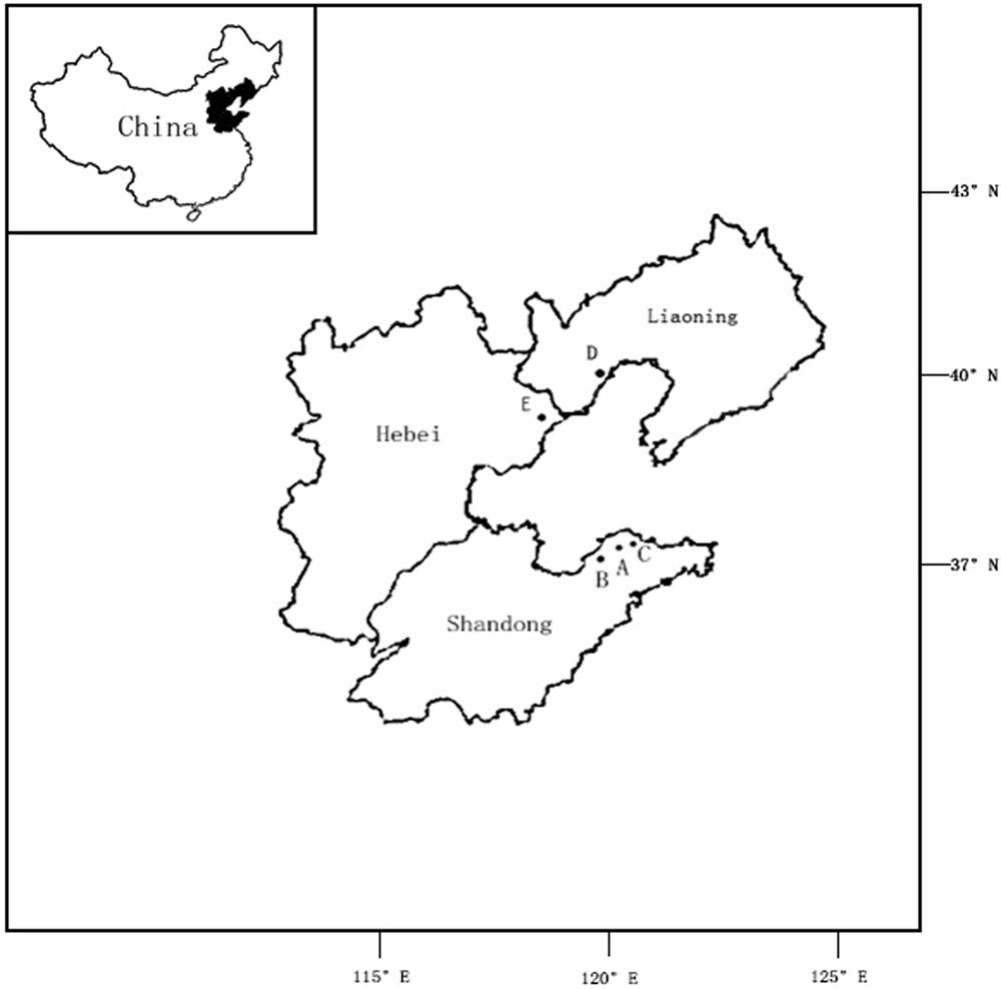
Location of sampling sites of soil samples around Bohai Gulf of China in five soil samples. The “A”, “B”, “C”, “D” and “E” refers to the five sampling sites: Qixia, Muping, Laizhou, Huludao and Changli, respectively. Map in this figure was generated by software Photoshop cc2015 linked with http://baoku.360.cn/soft/show/appid/104210242.

Rhizosphere soil samples of ATS and PAT were collected from orchards in five locations around the Bohai Gulf in China (Fig. 4, generated by Photoshop cc2015, http://baoku.360.cn/soft/show/appid/104210242): Qixia, Muping, and Laizhou in Shandong Province, Huludao in Liaoning Province, and Changli in Hebei Province. Samples were encoded with a combination of numbers and letters indicating I: location (A, Qixia; B, Muping; C, Laizhou; D, Laizhou; E, Changli), and II: soil type (1, soil from 25-year-old apple trees or PAT; 2, soil from five-year-old apple trees or ATS). Soil samples were randomly collected using the five-point sampling method. From each point, 1 kg of soil was collected within the root zone at a depth of 10–30 cm. Soils of the same type collected in the same plot were thoroughly mixed as one sample, placed in a sterilised bag, immediately placed on ice, and transported to the laboratory, where it was stored at −20 °C until physicochemical parameters measurement and DNA extraction. The physicochemical parameters of the soil were measured as follows. The oven dry-weight method allowed the estimation of soil moisture content. Soil pH was determined using a glass electrode meter (Sartorius PB-10, Sartorius scientific Instruments (Beijing) Co., Ltd.) in a suspension of 1 g of soil in 5 mL of distilled water. AP was extracted using sodium bicarbonate and then measured by the molybdenum blue method. AK was determined by flame photometry^32, 35^. AN was determined by potassium persulfate oxidation. OM content was determined as described by Walkey and Black^36, 37^.

### DNA extraction and PCR amplification

Microbial DNA was extracted from 0.5 g soil samples using the Power Soil DNA kit (MoBio Laboratories, Solana Beach, CA), following to the manufacturer’s protocols^38^. The V3–V4 regions of the bacterial 16S rRNA gene were amplified by polymerase chain reaction (PCR) (95 °C for 2 min, followed by 25 cycles of 95 °C for 30 s, 55 °C for 30 s, and 72 °C for 45 s, with a final extension at 72 °C for 10 min) using the primers^39^ 338 F 5′-barcode-ACTCCTACGGGAGGCAGCA-3′ and 806 R 5′-barcode-GGACTACHVGGGTWTCTAAT-3′^40^. The barcode is an eight-base sequence unique to each sample. The fungal 18S rRNA gene was amplified using PCR (98 °C for 2 min, followed by 35 cycles of 98 °C for 15 s, 56 °C for 30 s, and 72 °C for 40 s, with a final extension at 72 °C for 10 min) using the primers SSU0817F 5′-barcode-TTAGCATGGAATAATRRAATAGGA-3′ and SSU1196R 5′-barcode-TCTGGACCTGGTGAGTTTCC-3′^41^. PCRs were performed in triplicate in a 20-μL mixture containing 4 μL of 5 × Fast Pfu buffer, 2 μL of 2.5 mM deoxy-ribonucleoside triphosphate (dNTPs), 0.4 μL of each primer (5 μM), 0.4 μL of Fast Pfu polymerase, and 10 ng of template DNA.

### Illumina MiSeq sequencing

Amplicons were extracted from 2% agarose gels and purified using the AxyPrep DNA Gel Extraction Kits (Axygen Biosciences, Union City, CA, USA). Purified amplicons were pooled in equimolar and paired-end sequenced (2 × 300) on an Illumina MiSeq platform (Majorbio, Shanghai) according to the standard protocols.

### Processing and analysing of sequencing data

Raw FASTQ files were de-multiplexed and quality-filtered using QIIME(version 1.17) with the following criteria: (i) The 300 bp reads were truncated at any site with an average quality score of <20 bp over a 10 bp sliding window, and truncated reads shorter than 50 bp were discarded; (ii) exact barcode matching, two nucleotide mismatch in primer matching, and reads containing ambiguous characters were removed; and (iii) only overlapping sequences longer than 10 bp were assembled according to their overlapped sequence. Reads that could not be assembled were discarded. Operational taxonomic units (OTUs) with 97% similarity cutoff were clustered using UPARSE (version 7.1), and chimeric sequences were identified and removed using UCHIME.

Rarefaction analysis based on Mothur v.1.21.1^42^ was conducted to reveal diversity indices, including the Chao, abundance-based coverage estimator (ACE), and Shannon diversity indices. The beta diversity analysis was performed using UniFrac to compare the results of the principal component analysis (PCA) using the community ecology package, R-forge. Venn diagrams were implemented and Mantel test, redundancy analysis, and Heatmap figures were performed in Vegan packages in R.

### Sequence analysis

Data yielded from Illumina MiSeq sequencing was analysed with the bioinformatics platform Mothur (v.1.33.0), as described by Schloss *et al*.^42^.

After removing primers, all sequences were aligned with the Silva-ARB database. After chimeras were removed, all sequences were clustered into OTUs at a 97% identity threshold using the unsupervised Bayesian clustering algorithm CROP^43^, and all OTUs were included in downstream analyses. Shannon–Wiener curves of all the samples at a dissimilarity of 0.03 were generated. Rarefaction analysis suggested that the sequences sampled were sufficient to capture the total richness at a genetic distance of 0.03. To estimate the final ranking, we calculated the ACE and the estimated asymptotic microbial taxon richness Chao. After an identical number of reads were subsampled from each sample, microbial diversity was compared between PAT and ATS plants based on the calculated Shannon index. A graphical representation of the relative abundance of microbial diversity was created using Origin (version 8.0).

To ensure the data met the assumptions of normality, soil AN, soil AP, soil AK, soil moisture, and soil OM and pH were tested. Finally, redundancy analysis was used to identify the effect of environmental factors on bacterial and fungal community composition, based on the relative abundance of the detected phyla in each sample, with CANOCO 5.0 software.

## Electronic supplementary material

Dataset 1
