# Supplementary material for: Microbial community analysis of apple rhizosphere around Bohai Gulf
Source: Sci Rep. 2017 Aug 21;7:8918. doi: 10.1038/s41598-017-08398-9 (PMC5566992; doi:10.1038/s41598-017-08398-9)
Supplement: Supplementary file 1 — Dataset 1 [file 41598_2017_8398_MOESM1_ESM.doc]

**Microbial community analysis of apple**

**rhizosphere around Bohai Gulf**

**Jihang Jiang^1,*^, Zhen Song^2,*^, Xiaotong Yang^1^, Zhiquan Mao^3^, Xiaohong Nie^1^, Hui Guo^1,*,*^, Xiawei Peng^1,*,*^**

^1^College of Biological Sciences and Biotechnology, Beijing Forestry University, Beijing 100083, China.

^2^ Institute of Environment and Sustainable Development in Agriculture, Chinese Academy of Agricultural Sciences, Beijing, 100081, China

^3^ College of Chemistry and Material Science, Shandong Agricultural University, Taian, 271000, China

^*^These Authors contributed equally to the work

Correspondence and requests for materials should be addressed to G.H. (email: guohuiya@126.com)

# Supplementary Figures


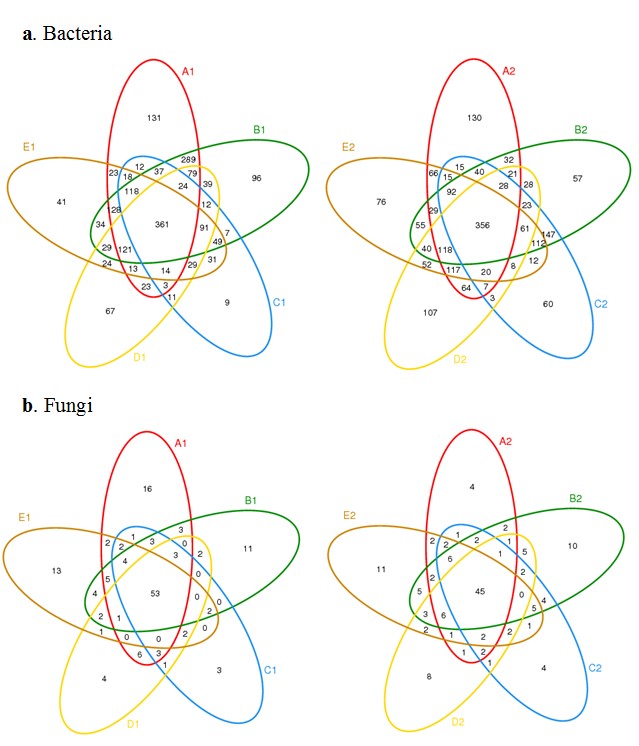


**Figure S 1.Venn Graph based on 97% similarity. The “A1”, “B1”, “C1”, “D1” and “E1” refers to the five PAT soils in** **Qixia, Muping, Laizhou, Huludao and Changli. The “A2”, “B2”, “C2”, “D2” and “E2” refers to the five ATS soils in Qixia, Muping, Laizhou, Huludao and Changli, respectively.**


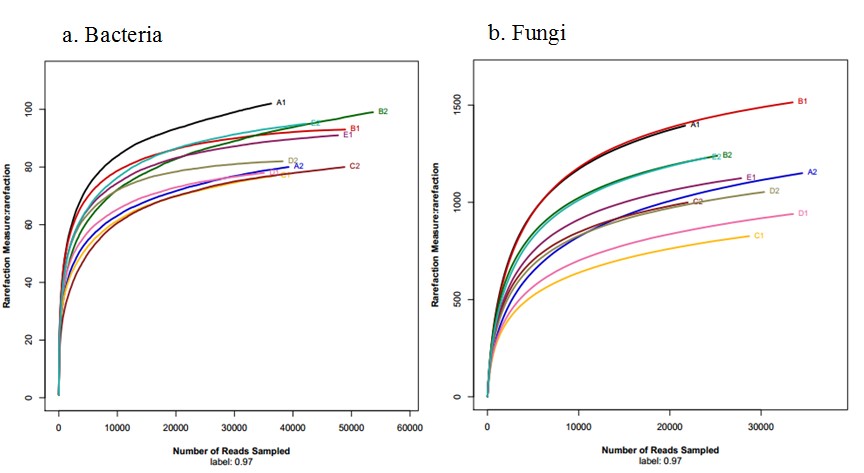


**Figure S 2.Rarefaction curve of bacterial and fungal depicting the effect of 3% dissimilarity on the number of OTUs identified in the 10 soil samples. The “A1”, “B1”, “C1”, “D1” and “E1” refers to the five PAT soils in** **Qixia, Muping, Laizhou, Huludao and Changli. The “A2”, “B2”, “C2”, “D2” and “E2” refers to the five ATS soils in Qixia, Muping, Laizhou, Huludao and Changli, respectively.**


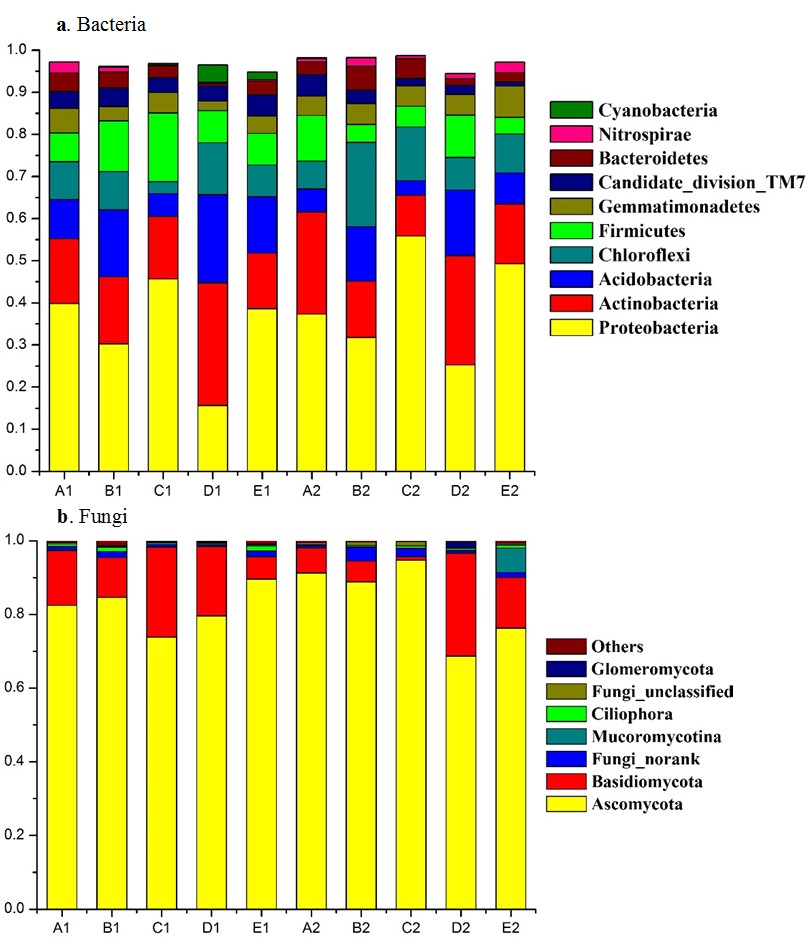


**Figure S 3.Relative abundance of main bacterial (a) and fungal (b) phylum in ten samples’ rhizosphere soil. The “A1”, “B1”, “C1”, “D1” and “E1” refers to the five PAT soils in** **Qixia, Muping, Laizhou, Huludao and Changli. The “A2”, “B2”, “C2”, “D2” and “E2” refers to the five ATS soils in Qixia, Muping, Laizhou, Huludao and Changli, respectively.**
